# Modulation of intestinal metabolites by calorie restriction and its association with gut microbiota in a xenograft model of colorectal cancer

**DOI:** 10.1007/s12672-024-00897-2

**Published:** 2024-02-22

**Authors:** Yuhuan Zhang, Lintao Dong, Xingchen Dai, Yongli Huang, Yujing Gao, Fang Wang

**Affiliations:** 1 Department of Gastroenterology, General Hospital, Ningxia Medical University, 804 Shengli South Street, Yinchuan, 750004 Ningxia People’s Republic of China; 2 School of Clinical Medicine, Ningxia Medical University, Yinchuan, China; 3 Key Laboratory of Fertility Preservation and Maintenance of Ministry of Education, Department of Biochemistry and Molecular Biology, School of Basic Medical Sciences, Ningxia Medical University, 1160 Shengli Street, Yinchuan, 750004 Ningxia People’s Republic of China; 4 National Health Commission Key Laboratory of Metabolic Cardiovascular Diseases Research, Ningxia Medical University, Yinchuan, 750004 Ningxia People’s Republic of China

**Keywords:** Caloric restriction, Colorectal cancer, Intestinal metabolites, Gut microbiota

## Abstract

**Background:**

Colorectal cancer (CRC) is a common malignant tumor, and its occurrence and development are closely related to dysbiosis of gut microbes. Previously, we found calorie restriction altered the composition of the microbial community in a colorectal cancer mouse model and inhibited in vivo growth of CRC cells. Here, we aim to further investigate alteration in the intestinal metabolites and explore the interplay between gut microbiota and intestinal metabolites upon calorie restriction.

**Methods:**

Human colorectal cancer HCT116 cells were used to establish a colorectal cancer xenograft mouse model. The changes of intestinal metabolites in the ad libitum group and calorie restriction group were investigated through untargeted metabolomics analysis. The integrative analysis of gut microbiota and metabolites to elucidate the associations between gut microbiota and intestinal metabolites.

**Results:**

Compared with the mice in the ad libitum group, mice upon calorie restriction exhibited downregulation of Isoleucyl-Valine, and upregulation of D-Proline, 1-Palmitoylphosphatidylcholine, and 4-Trimethylammoniobutanoic acid. Additionally, an integrative analysis of gut microbiota and metabolites revealed that *Lactobacillus, Parabacteroides* and *rC4-4* genus were upregulated in the calorie restriction group and positively correlated with D-Proline, 4-Trimethylammoniobutanoic acid or 1-Palmitoylphosphatidylcholine, while negatively correlated with Isoleucyl-Valine. In contrast, the *Nitrospirae* and *Deferribacteres* phylum exhibited opposite trends.

**Conclusion:**

Calorie restriction affects the abundance of gut microbes such as *Nitrospirae* phylum and *Lactobacillus* genus in mouse model of colorectal cancer, leading to changes in the metabolites such as D-Proline、Isoleucyl-Valine, which contributes to the suppression of in vivo growth of CRC by calorie restriction.

## Introduction

Colorectal cancer (CRC) ranks third in terms of incidence (10.0%) and second in terms of mortality (9.4%) among all cancers [1]. Nutrition, environment, and lifestyle are all relevant risk factors for the development of colorectal cancer, with high-calorie diets and consumption of red and processed meats increasing the risk of colon and rectal cancer [2]. Additionally, the incidence of colorectal cancer is closely associated with the Human Development Index (HDI), with a continuous increase in the incidence of colon and rectal cancer observed in most countries with high and medium HDI levels, as well as among young individuals [3].

Caloric restriction (CR), which involves reducing total daily intake by 20%-40%, has been shown to have beneficial effects such as reducing inflammation, promoting autophagy, enhancing stress resistance, and modulating the gut microbiota [4]. The fact that calorie restriction can delay cancer was first described in animal models in the 1980s [5]. By altering dietary patterns, particularly by reducing factors that promote colorectal cancer development such as high-fat, high-cholesterol, high-red meat, and low-fiber diets, the risk of colorectal cancer can be reduced [6]. However, the precise mechanisms underlying the impact of calorie restriction on colorectal cancer remain unclear.

The impact of the gut microbiota on human health and disease has been receiving increasing attention, and numerous studies have demonstrated a close association between gut microbiota and cancer development and treatment [7, 8]. Our previous study indicated that calorie restriction alters the composition of the gut microbiota in a CRC mouse model, resulting in an increase in beneficial bacteria such as *Parabacteroides*, *Bacteroides*, *Roseburia*, and *Lactobacillus*, consequently affecting CRC cells proliferation [9]. The gut microbiota is capable of producing various bioactive metabolites which are absorbed into the host’s bloodstream and enter the enterohepatic circulation. Through its influence on host metabolism, the gut microbiota plays a crucial role in the occurrence and progression of CRC [10].

Cancer cells often exhibit increased activity in the glycolytic pathway, a phenomenon known as the Warburg effect [11]. This abnormal metabolic pathway leads to enhanced conversion of glucose into lactate. Butyrate, a short-chain fatty acid (SCFA) produced from dietary fiber, has been shown to inhibit CRC growth in a dose-dependent manner [12]. CRC cells exhibit increased uptake and utilization of amino acids, with specific amino acids like glutamate, glutathione, and arginine playing important parts in cancer cell growth and survival [13]. These findings highlight the importance of gut microbiota and metabolic changes in CRC development. Here, based on our previous study, we further explored the changes in the intestinal metabolites in response to caloric restriction in a xenograft model of colorectal cancer. Moreover, we conducted an integrated analysis of metabolomics and gut microbiota to explore potential mechanisms by which caloric restriction suppresses CRC cell growth.

## Materials and methods

### Reagents and cell culture

The human colorectal cancer HCT116 cells were obtained from Wuhan Procell Life Science & Technology Co., Ltd. (cat. no. CL‑0096). HCT116 cells were cultured in RPMI‑1640 medium (cat. no. AG29714278, Hyclone, Cytiva) supplemented with 10% FBS (cat. no. 11011‑8611, Zhejiang Tianhang Biotechnology Co., Ltd.) and 1% penicillin–streptomycin ((cat. no. ST488, Beyotime Institute of Biotechnology) in a 5% CO_2_ incubator at 37 ℃. When the cells reached 80–90% confluence, the cells were digested with 0.05% trypsin cell digestion solution (cat. no. C0202, Beyotime Institute of Biotechnology) at 37 °C for about 1 min, and the subcultures were continued.

### Animals

Animal experiment was approved by the Ningxia Medical University Ethics Committee (approval no. 2021–045). A total of 10 specific-pathogen-free grade male BALB/c nude mice (age, 4 weeks; body weight, 18–20 g) were acquired from Beijing Vital River Laboratory Animal Technology Co., Ltd. Mice were raised in the temperature 22 ± 1 °C, humidity 40–60% (SPF environment) and 12/12-h light–dark cycle environment of the Experimental Animal Center of Ningxia Medical University. All mice were guaranteed an adequate supply of water and food.

### Mouse model of colorectal cancer xenograft tumor and diet treatment

2 × 10^6^/100 µl HCT116 cells were injected into the subcutaneous area of the right flank of 10 nude mice after 1 week of adaptation feeding. The weight of the food consumed by the mice, tumor size and body weight were measured every other day. The tumor volume was calculated using the formula a^2^ × b × 0.5, where a is the shortest diameter and b is the diameter perpendicular to a [14]. When the average tumor volume of each nude mouse reached 100 mm^3^ (12 days after tumor inoculation), the mice were randomly divided into ad libitum feeding group and a calorie restriction (CR) group and each group consisted of 5 animals. The weight of the food consumed, tumor size, body weight, feeding habits, and physical activity were tracked every other day. After 3 weeks, the mice were euthanized by inhalation of CO_2_ gas (20% of the euthanasia chamber volume/min as a controlled flow rate of CO_2_, which was increased to 100% of the euthanasia chamber volume/min once the mice were unconscious). Mouse xenograft tumors and intestinal contents (solid excreted feces or collected from the rectum and a small portion of semi‑solid stool with relatively abundant water content that was at the end of the colon) were collected.

### Fecal sample preparation

The intestinal contents of mice were collected after execution and metabolomics studies were conducted at Shanghai Luming Biotechnology Co., LTD., Shanghai, China. All experiments involving animals were performed in compliance with the guidelines for the ethical review of laboratory animal welfare People’s Republic of China National Standard (GB/T 35892-2018), adhering to the ethical standards and regulations for animal research. All laboratory procedures and safety measures were rigorously followed as per the guidelines set forth by safety management regulations of Ningxia Medical University, ensuring the highest standards of biosafety and laboratory safety. Quality control samples (QC) were prepared by mixing the extracts of all samples in equal volumes. 30 mg of sample was weighed and loaded into 1.5 mL EP tubes. Add two small steel beads to the tube. A mixture of 300 μl methanol and water (V: V = 4:1 containing L-2-chlorophenylalanine, 4 μg/mL) was added to each sample. The samples were precooled in the refrigerator at – 40 °C for 2 min and then ground in a grinder (60 Hz, 2 min). Then the samples were extracted by ultrasound in an ice water bath for 10 min and left at – 40℃ for 30 min. The extract was centrifuged at 4 °C (13000 rpm) for 10 min, and 200 μl of the supernatant was taken to dry in a frozen concentration centrifuge dryer. A mixture of 300 μl methanol and water (V: V = 1:4) was added to each sample, followed by vortexing for 30 s and sonication for 3 min. The samples were allowed to stand for 2 h at – 40 °C. Samples were centrifuged for 10 min at 4 °C (13,000 rpm), and 150 μl of the supernatant was aspirated by syringe, filtered using 0.22 μm microfilters, transferred to LC injection vials, and stored at – 80 °C until LC–MS analysis.

### LC–MS/MS analysis

Using the ACQUITY UPLC I-Class system (Waters Corporation,Milford, USA) with Q-Exactive plus quadrupole-Orbitrap mass spectrometer equipped with heated electrospray ionization(ESI) source (Thermo Fisher Scientific, Waltham, MA, USA) metabolic profiles were analyzed in ESI positive/negative ion mode. An ACQUITY UPLC HSS T3 column (1.8 μm, 2.1 × 100 mm) were employed in both positive and negative modes. The binary gradient elution system consisted of (A) water (containing 0.1% formic acid, v/v) and (B) acetonitrile (containing 0.1% formic acid, v/v). Linear gradient is set as follows: 0 min, 5% B; 2 min, 5% B; 4 min, 30% B; 8 min, 50% B; 10 min, 80% B; 14 min, 100% B; 15 min, 100% B; 15.1 min, 5% B and 16 min, 5%B. The flow rate was 0.35 mL/min and column temperature 45 ℃. All the samples were kept at 4 ℃ during the analysis. The injection volume was 2 μL.

### Data preprocessing and analysis

The original LC–MS data was processed using PronenesisQIV2.3 software (Nonlinear, Dynamic, Newcastle, UK) for baseline filtering, peak recognition, integration, retention time correction, peak alignment and normalization. Qualitative analysis was performed using human metabolome database (HMDB, http://www.hmdb.ca), lipid map (V2.3), Metlin, EMDB, PMDB and self-built database. Orthogonal partial least squares discriminant analysis (OPLS-DA) and partial least squares discriminant analysis (PLS-DA) were used to identify the metabolites that were different between groups. To prevent overfitting, sevenfold cross-validation and 200 Response Permutation Testing (RPT) were used to evaluate the quality of the model. According to the OPLS-DA model, the projected variable importance (VIP) values of the metabolites were ranked to assess the overall contribution of each variable to population differences. A two-tailed Student’s T-test was further used to verify whether the metabolites of difference between groups were significant. Differential metabolites with VIP value greater than 1.0 and p value less than 0.05 were screened. Using Pearson rank correlation analysis and calculation of the correlation between metabolites. Differences metabolites using Kyoto Encyclopedia of Genes and Genomes (KEGG, http://www.genome.jp/kegg) database for pathway enrichment analysis.

### Microbiota and metabolites analysis

Fecal microbiota analysis was investigated as previously described [9]. After quality control which included the steps of primer removal, quality filtering, denoise, stitching, and removal of chimerism, the data were evaluated for bacterial species differences using the QIIME2 (version 2019.4) gene cloud platform(https://www.genescloud/). Selection of species abundance before 20 for subsequent analysis. Using Spearman rank correlation analysis and calculation of the correlation between microbiota and metabolites.

### Statistical analysis

Statistical analyses for quantitative multiple group comparisons were performed using Student’s t-test, analysis of variance (ANOVA), or Wilcoxon test. Spearman grade correlation analysis is performed for substances with significant differences in the original omics to calculate correlations between microbiota and metabolites. When *P* < 0.05, the difference was considered significant.

## Results

### Establishment of colorectal cancer xenograft and calorie restriction model in mice

**Fig. 1:**
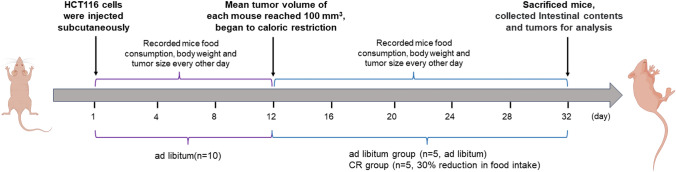
Establishment of colorectal cancer xenografts and calorie restriction models in mice

We plotted colorectal cancer xenograft and calorie restriction mouse model by Figdraw (Fig. 1). Twelve days after subcutaneous injection of HCT116 cells, mice were randomly divided into ad libitum group and CR group, in which CR group was restricted to 30% diet. After 3 weeks, mouse tumors were collected and weighed. The weight loss of the CR group mice was not significant compared with the ad libitum feeding group after the calorie restriction to the mice, and the results were not statistically significant. However, CR significantly suppressed tumor volume and tumor compared with the control group on the 32nd day [9].

### Calorie restriction alters intestinal metabolites in the xenograft model of CRC

**Fig. 2:**
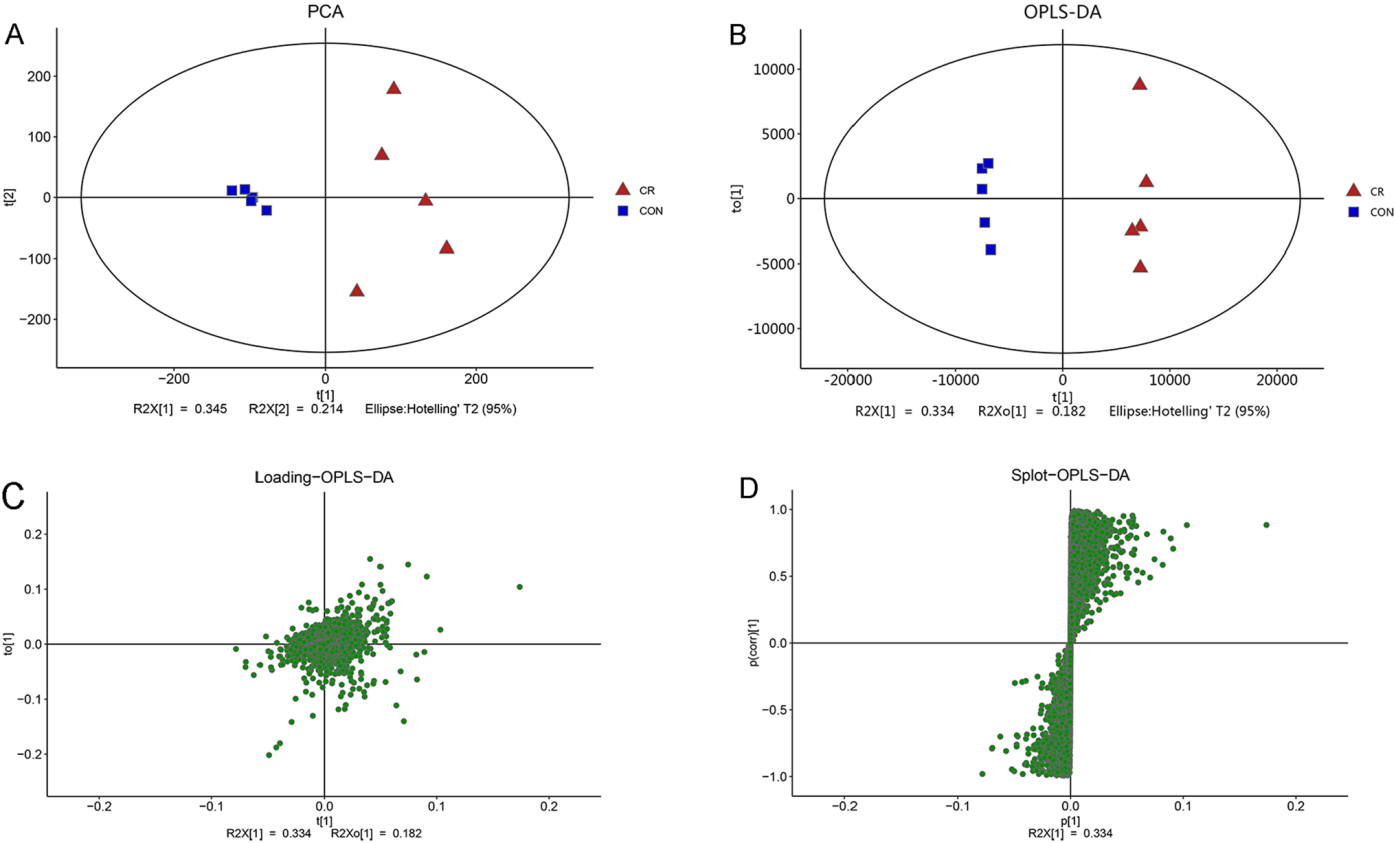
Intestinal metabolites of mice with colorectal cancer were analyzed by LC–MS/MS. **A**, **B** PCA and OPLS-DA scores plot of metabolites in mice between ad libitum (Control) group and calorie restriction (CR) group were analyzed by principal component analysis. **C**, **D** Loading analysis and S-PLOT analysis of colorectal cancer mice metabolites

Previously, we established a xenograft model of CRC using HCT116 cells to investigate the impact of calorie restriction on in vivo growth of CRC cells, and found calorie restriction could remarkably reduce development of CRC. Importantly, calorie restriction substantially remodels gut microbiota of the mice. To further explore the possible mechanism by which calorie restriction suppresses CRC, we analyzed the intestinal metabolites alteration through LC–MS/MS analysis. The PCA score plot demonstrated distinct distribution patterns of metabolites between the mice in the ad libitum group and calorie restriction group (Fig. 2A). Furthermore, the OPLS-DA model revealed significant differences between the two groups, with all sampling points falling within the 95% confidence interval (Fig. 2B). Additionally, the OPLS-DA Loading plot and S-plot demonstrated significant changes in metabolites that were distant from the axis (Fig. 2C, D). These findings suggest calorie restriction significantly alters the intestinal metabolites levels of the CRC tumor-bearing mice.

### Identification of differential intestinal metabolites in the calorie-restricted mice

**Fig. 3:**
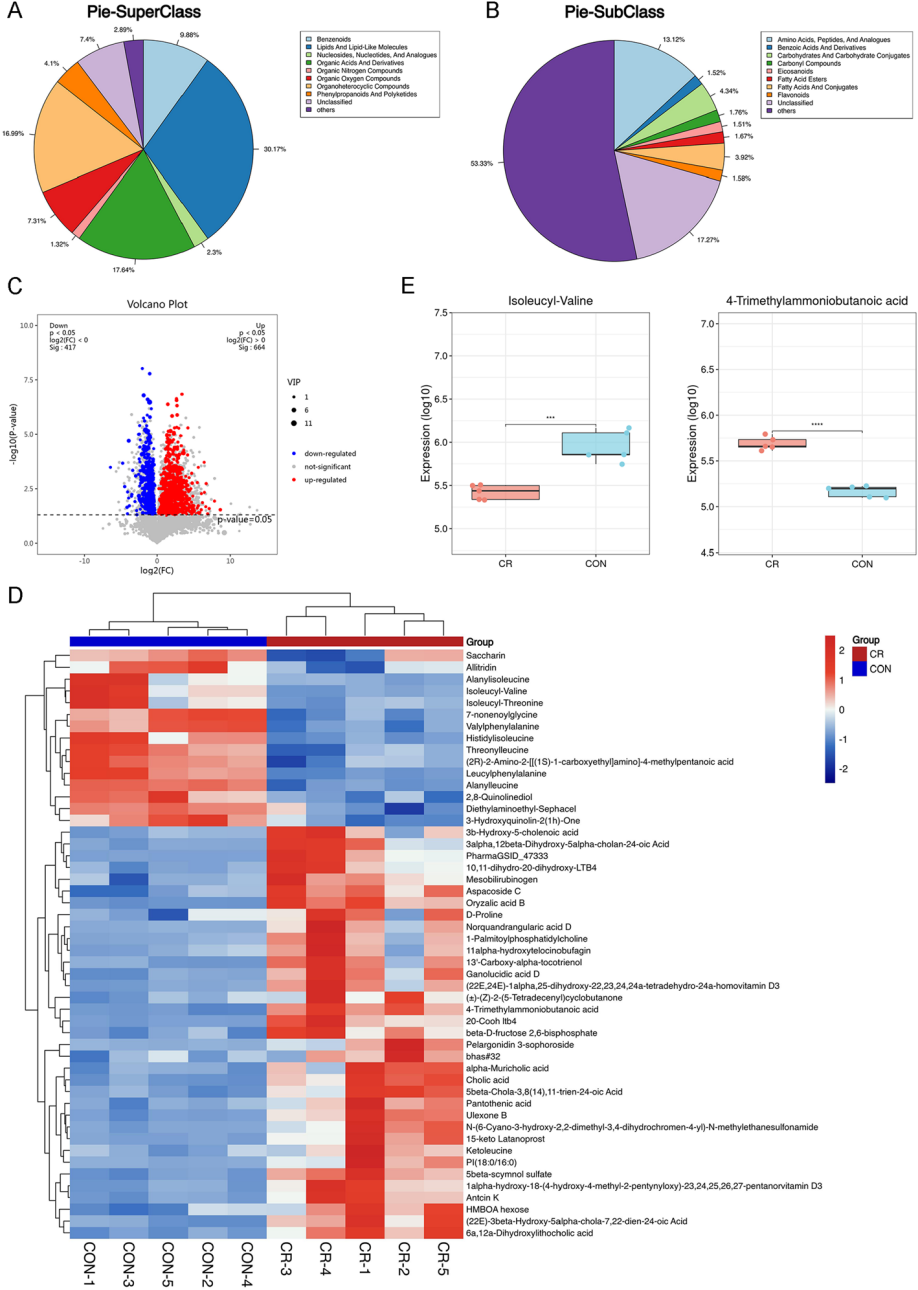
Identification of differential intestinal metabolites in the calorie-restricted mice. **A**,** B** Super-Class and Sub-Class types and percentages of metabolites of mice intestinal metabolites. **C** Volcanic map of up—and down-regulated expression of differential metabolites. Blue dots represent down-regulated differentially expressed metabolites, red dots represent up-regulated differentially expressed metabolites, and gray dots represent detected but not significantly different metabolites. **D** Heatmap of the differential metabolites in ad libitum (Control) group and calorie restriction (CR) group. **E** Box plots showing the levels of Isoleucyl−Valine and 4-Trimethylammoniobutanoic in ad libitum (Control) group and calorie restriction (CR) group. Data are presented as mean ± SD, n = 5. *P < 0.05, ***P < 0.001

Next, we analyzed the differential metabolites between the mice in the ad libitum group and calorie restriction group. As demonstrated in Fig. 3A, the Super-class of metabolites in the CRC tumor-bearing mice consist of Lipids and lipid-like molecules, Organic acids and derivatives, and Organoheterocyclic compounds. Sub-class such as Amino acids, peptides and analogues, Carbohydrates and carbohydrate conjugates, and Fatty acids and conjugates primarily constitute the classification of metabolites (Fig. 3B). A combination of multidimensional analysis and single-dimensional was employed to screen for differentially expressed metabolites between groups. Using criteria of VIP > 1 and *P* < 0.05, a total of 1081 differentially expressed metabolites were detected, with 664 metabolites upregulated and 417 metabolites downregulated (Fig. 3C).

We generated a heatmap (Fig. 3D) based on the expression levels of the top 50 significantly different metabolites ranked by VIP score. In the heatmap, it can be observed that metabolites such as Saccharin, Isoleucyl-Valine, Histidylisoleucine, 7-nonenoylglycine, and Alanylleucine were downregulated in the CR group. However, metabolites including 3b-Hydroxy-5-cholenoic acid, D-Proline, 1-Palmitoylphosphatidylcholine, 11alpha-hydroxytelocinobufagin, 13′-Carboxy-alpha-tocotrienol, Ganolucidic acid D, 4-Trimethylammoniobutanoic acid, Pantothenic acid, Ketoleucine, 5beta-scymnol sulfate, Antcin K, and HMBOA hexose showed upregulation in the CR group. Additionally, according to the VIP sorted, box plots of the up-regulated Isoleucyl − Valine (*P* < 0.05) and the down-regulated differential metabolites 4 − Trimethylammoniobutanoic acid (*P* < 0.05) were also depicted in Fig. 3E.

### Correlation analysis of the differential intestinal metabolites

**Fig. 4:**
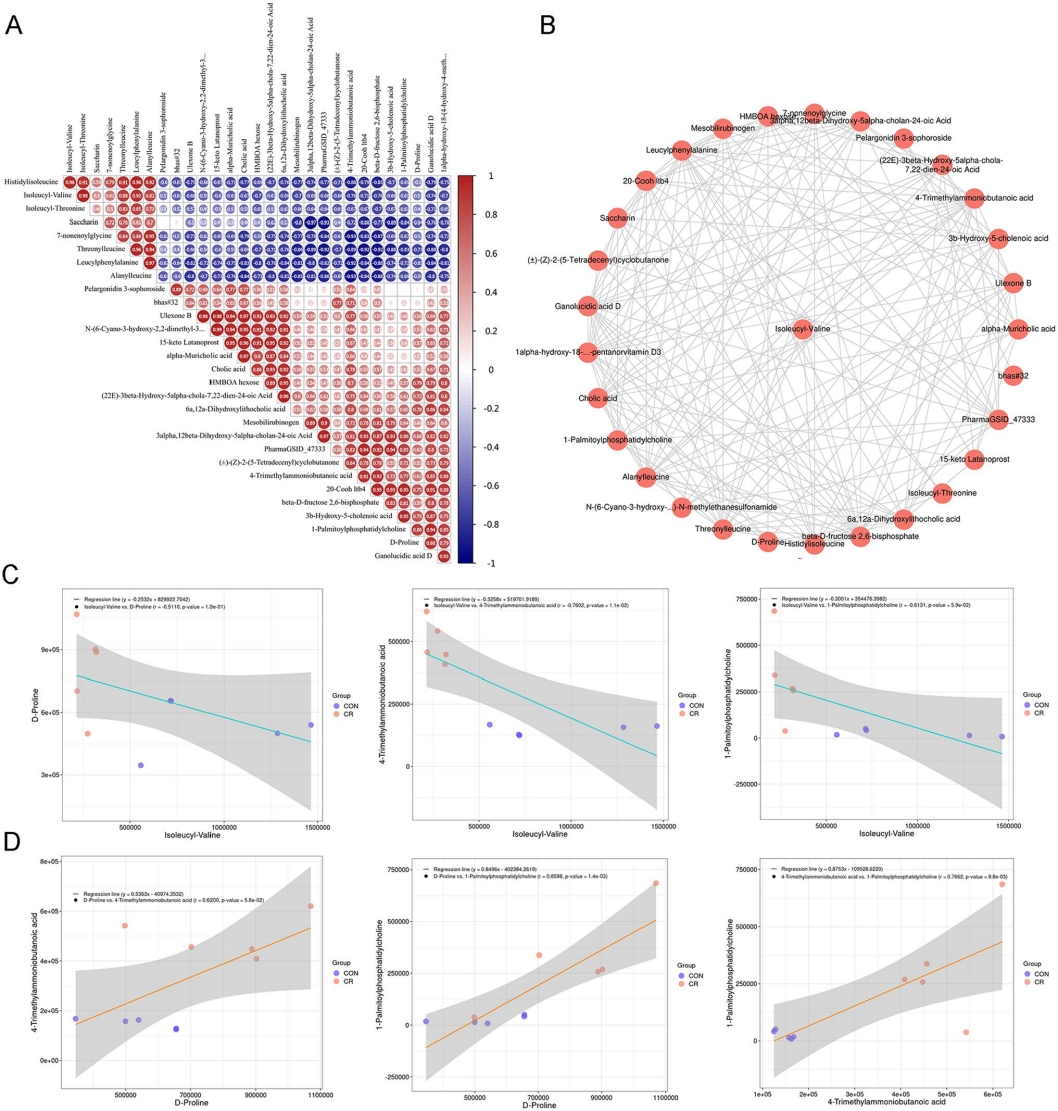
Correlation analysis of the differential intestinal metabolites. **A** Pearson correlation analysis of fecal microbiota and metabolites. p-values are depicted from blue to red, where red represents positive correlations and blue represents negative correlations. **B** Network map of top 30 differential metabolites. **C**, **D** Scatter plots of negative and positive correlations among some metabolites. Purple dots represent the ad libitum feeding group, red dots represent the CR group, and r is the correlation coefficient

We performed a correlation analysis on the top 30 metabolites ranked by VIP score. The correlation plot revealed that Isoleucyl-Valine is positively correlated with Histidylisoleucine and Alanylleucine, while negatively correlated with 4-Trimethylammoniobutanoic acid. Conversely, 4-Trimethylammoniobutanoic acid is negatively correlated with Histidylisoleucine and Alanylleucine, but positively correlated with 1-Palmitoylphosphatidylcholine. Furthermore, 1-Palmitoylphosphatidylcholine is positively correlated with D-Proline and negatively correlated with Histidylisoleucine and Alanylleucine (Fig. 4A, C, D). The correlation analysis indicates the presence of metabolic networks among the primary metabolites (Fig. 4B). Additionally, these metabolites may interact through different metabolic pathways, thereby exerting distinct effects.

### Enrichment analysis of metabolic pathways of differential metabolites

**Fig. 5:**
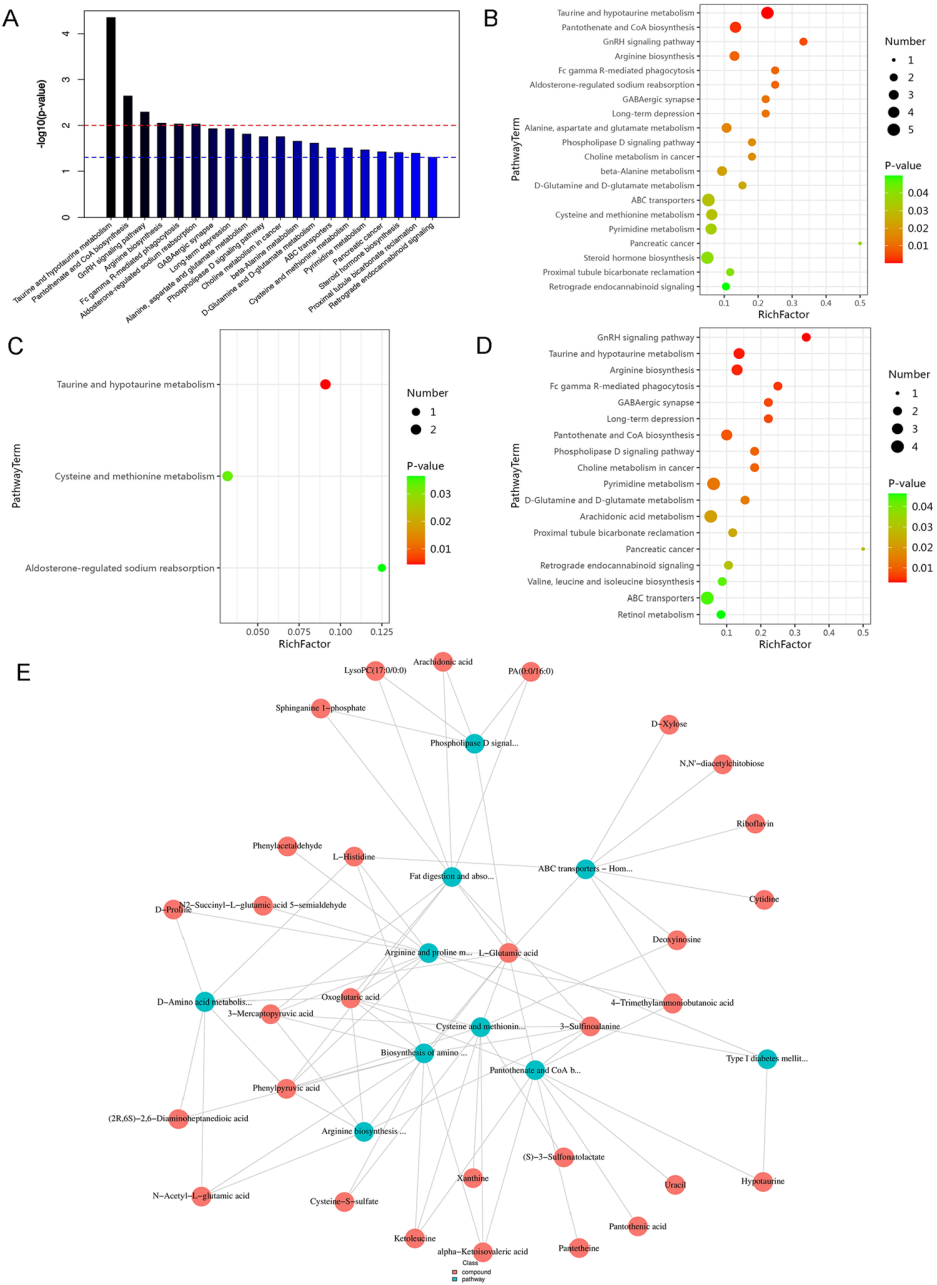
Metabolic pathways modulated by calorie restriction in the calorie-restricted mice. **A** Enrichment map of all differential metabolic pathways. The horizontal axis of the differential metabolite histogram represents the differential metabolic pathway, and the vertical axis represents p-value. **B** KEGG enriched bubble diagram. The horizontal axis of the KEGG enrichment bubble diagram represents the enrichment factor and the vertical axis represents the differential metabolic pathway. **C**, **D** Bubble diagram of down-regulated and up-regulated metabolic pathways. **E** A network diagram of metabolites and metabolic pathways

We next analyzed the differences metabolites, and explored the function of which metabolic pathways through Differential Metabolite Enrichment Analysis. The KEGG database was used to analyze the biological pathways associated with calorie-restricted metabolites. A total of 20 significantly enriched pathways were identified (*P* < 0.05). The top four metabolic pathways were Taurine and hypotaurine metabolism, Pantothenate and CoA biosynthesis, GnRH signaling pathway, and Arginine biosynthesis (Fig. 5A, B). Specifically, from the bubble diagram of Fig. 5C, Taurine and hypotaurine metabolism, Cysteine and methionine metabolism, and Aldosterone-regulated sodium reabsorption were downregulated in calorie restriction group. In contrast, Arginine biosynthesis, Pantothenate and CoA biosynthesis, Choline metabolism in cancer, Pyrimidine metabolism, D-glutamine and D-glutamate metabolism, Arachidonic acid metabolism, and Valine, leucine, and isoleucine biosynthesis were upregulated in calorie restriction group of Fig. 5D. In addition, we performed metabolome-FELLA enrichment analysis to analyze the relationship between metabolites and metabolic pathways [15]. Both D-Proline and 4-Trimethylammoniobutanoic acid are involved in the arginine and proline metabolism pathway (Fig. 5E). Furthermore, among the differentially regulated metabolites we detected, Isoleucyl-Valine is a metabolic product of the Valine, leucine, and isoleucine biosynthesis pathway.

### CR alters the composition of gut microbiota and metabolites

**Fig. 6:**
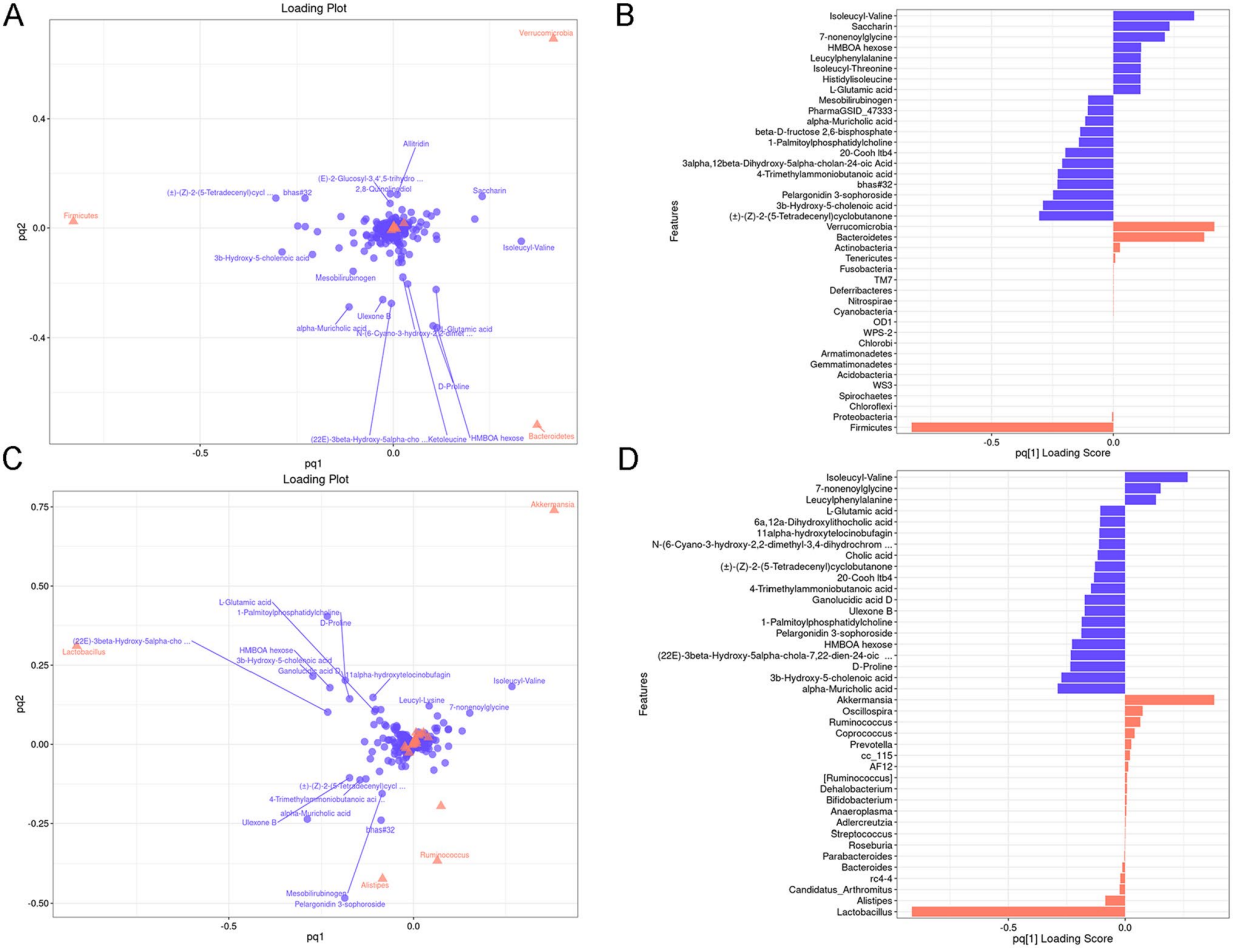
CR alters the composition of gut microbiota and metabolites. **A**, **C** Gut microbes and metabolites O2PLS loading plot at the phylum and genus level. Red represents gut microbes and purple represents metabolites. The absolute value of the load value indicates the strength of the association between gut microbes and metabolites, the greater the absolute value, show that relevance is the stronger. **B**, **D** Gut microbes and metabolites O2PLS feature importance sort diagram at the phylum and genus level. The higher the columns show that the greater importance

To determine the extent of their association and to identify the main microorganisms and metabolites that cause such association. Two-way Orthogonal Partial Least Squares (O2PLS) was used to perform bidirectional modeling and prediction in gut microbes and metabolites to mine the internal links between them. According to the O2PLS sort of important features diagram, *Verrucomicrobia*, *Bacteroidetes* and *Firmicutes* at the phylum level were the most important in gut microbiota and Isoleucyl-Valine and other metabolites are closely related to them (Fig. 6A, B). Besides, at the genus level, *Lactobacillus*, *Akkermansia* and *Alistipes* showed a close relationship with Isoleucyl-Valine, D-Proline and other metabolites (Fig. 6C, D).

### Integrative analysis of fecal metabolites and gut microbiota

**Fig. 7:**
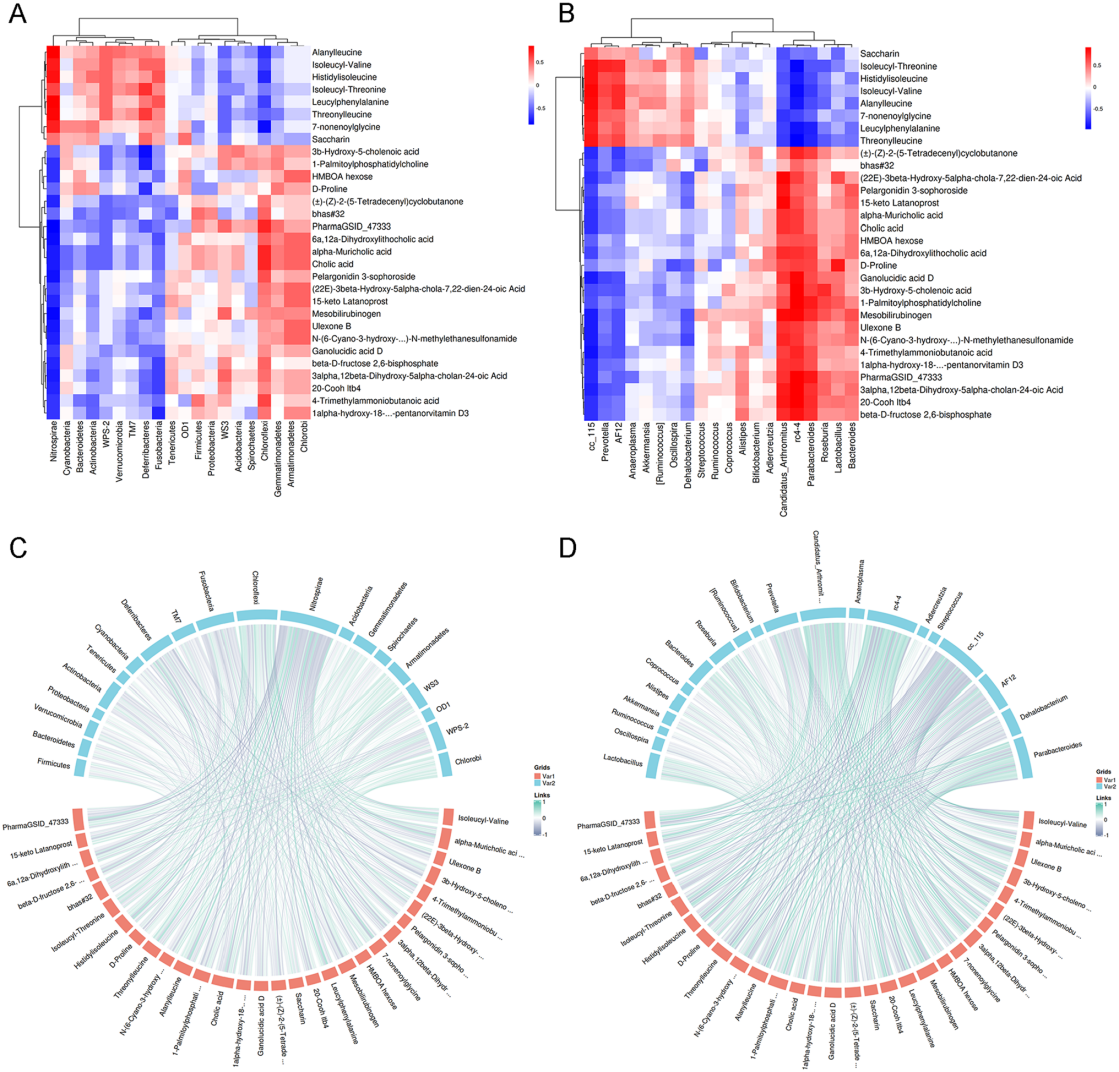
Integrative analysis of fecal metabolites and gut microbiota. **A**, **B** Correlation heatmaps between differential microbes (top 20 in abundance) and differential metabolites (top 30 in VIP) at the phylum and genus level. **C**, **D** Chord diagram of correlation between differential microbes (top 20 in abundance) and differential metabolites (top 30 in VIP) at the phylum and genus level. Red variable1 represents metabolites and blue variable2 represents the microbiota

In our previous study, 16S ribosomal RNA sequencing data revealed that calorie restriction reshaped the gut microbiota in the mice model of colorectal cancer. The gut microbiota plays a vital role in the breakdown and metabolism of various dietary components, producing a wide range of metabolites through their enzymatic activities. To investigate the potential influence of gut microbiota on metabolites, we conducted correlation analysis to assess the relationship between the distribution of gut microbial communities and metabolomic profiles. To visualize the correlations between gut microbiota and metabolites, we generated a correlation heatmap. Specifically, we selected the top 20 differentially abundant gut microbiota and the top 30 VIP-ranked differentially regulated metabolites for this analysis. At the phylum level, we observed positive correlations between *Nitrospirae* and metabolites such as Isoleucyl-Valine and Histidylisoleucine. However, *Nitrospirae* showed negative correlations with metabolites including 1-Palmitoylphosphatidylcholine, Ganolucidic acid D, 4-Trimethylammoniobutanoic acid and HMBOA hexose. Additionally, *Deferribacteres* exhibited negative correlations with 1-Palmitoylphosphatidylchol and D-Proline (Fig. 7A). At the genus level, *Lactobacillus* is negatively correlated with D-Proline; *Parabacteroides* and *rC4-4* displayed positive correlations with metabolites such as D-Proline, 4-Trimethylammoniobutanoic acid, 1-Palmitoylphosphatidylcholine, 3b-Hydroxy-5-cholenoic acid, and Ganolucidic acid D, while exhibiting negative correlations with Isoleucyl-Valine and Histidylisoleucine (Fig. 7B). Inaddition to the correlation heatmap, the same result was obtained from the correlation chord diagram between microbes and metabolites (Fig. 7C, D).

Collectively, these findings revealed that gut microbial taxa, such as *Lactobacillus*, could potentially play a role in modulating the metabolic pathways influenced by caloric restriction in the CRC mouse model. These microbial taxa may contribute to changes in relevant metabolites, which in turn could have implications for CRC tumorigenesis.

## Discussion

Restricting calorie intake can help maintain a healthy body weight and prevent excessive obesity [16]. CR inhibited tumor size in the mouse model of CRC xenograft in our previous study [9], but had no significant effect on the weight of the mice. Meanwhile, immunohistochemical staining of CRC tumor tissue in mice displayed that CR restrained the proliferation of CRC and promoted its apoptosis, thus confirming the conclusion that CR suppresses the growth of CRC in vivo. Studies have shown that caloric restriction (CER) inhibits spontaneous tumors in p53-deficient mice [5, 17], prolongs the lifespan of rhesus monkeys and reduce the occurrence of spontaneous cancer in them [18, 19]. Limiting the consumption of red meat, processed meat, dietary fat, and consuming a high-fiber diet can to some extent reduce the risk of colorectal cancer [2]. However, our understanding of the relationship between calorie restriction and colorectal cancer is still limited.

In our previous research, we found that calorie restriction inhibits the proliferation of CRC cells and can alter the abundance of gut microbiota [9]. The gut microbiota plays a role in the breakdown of dietary fiber and other indigestible substances, producing various metabolites. The levels of SCFAs are decreased in the feces of colorectal cancer patients [20, 21], which may be related to a reduction in bacteria that produce *butyrate*, *Bacteroides vulgatus* and *Roseburia* [22–24]. In obese individuals, there is a significant decrease in the abundance of *Bacteroides thetaiotaomicron*, a butyrate-producing bacterium, which is negatively correlated with serum glutamate concentrations [25].

In present study detected that calorie restriction can alter the composition of gut metabolites. Metabolites such as Isoleucyl-Valine were downregulated, while D-Proline, 4-Trimethylammoniobutanoic acid, and 1-Palmitoylphosphatidylcholine were upregulated. These metabolites may be associated with upregulated metabolic pathways such as Arginine and proline metabolism, Valine*,* leucine*,* and isoleucine biosynthesis, and Choline metabolism in cancer. Leucine, isoleucine, and valine are components of branched-chain amino acids (BCAAs). Decreasing levels of isoleucine or valine have been shown to be effective in treating and preventing obesity and diabetes [26]. BCAAs can also influence the occurrence and progression of diseases through interactions with gut microbiota. Qiao et al. demonstrated that *Parabacteroides merdae* in the gut enhances BCAA catabolism to prevent obesity-related atherosclerosis [27].

Proline is a non-essential amino acid that primarily functions through the metabolic pathways of arginine and ornithine. In comparison to the normal control group, the concentration of D-proline in the plasma of Alzheimer’s disease rats is significantly reduced [28]. Levels of D-serine and D-proline serve as appropriate predictive factors for antidepressant response in major depressive disorder and post-traumatic stress disorder, as well as prognostic biomarkers for early cognitive decline [29]. In patients with inflammatory bowel disease (IBD) who experience fatigue symptoms, alterations in serum proline and fecal microbiota have been observed [30].

4-Trimethylammoniobutanoic acid (TMABA), also known as betaine, is a metabolite in dietary phospholipid choline metabolism. Betaine has shown positive effects in the management of type 2 diabetes [31], primarily by regulating genes related to non-alcoholic fatty liver disease (NAFLD) [32]. Additionally, supplementation with betaine has been found to ameliorate gut dysbiosis induced by a high-fat diet (HFD) and improve obesity and metabolic syndrome. This beneficial effect is mediated, at least in part, through the regulation of the miR-378a/YY1 signaling axis derived from the gut microbiota [33]. 0.1-Palmitoylphosphatidylcholine (PC) is a phospholipid that is abundantly present in biological organisms, particularly in cell membranes. A study has found an association between higher levels of PC and lower risk of conventional adenomas [34].

Calorie restriction reshapes the gut microbiota, leading to a reduction in the proportion of *Nitrospirae* phylum and *Deferribacteres* phylum, while increasing the abundance of *Lactobacillus*, *Parabacteroides* and *rC4-4* at the genus level. Through a combined analysis of microbiota and metabolomics, we identified correlations between specific microbial groups and metabolites. *Nitrospirae* phylum and *Deferribacteres* phylum displayed a positive correlation with *Isoleucyl-Valine* but a negative correlation with *4-Trimethylammoniobutanoic acid*. *Nitrospirae* phylum is significantly increased in patients with distal cholangiocarcinoma (dCCA) [35]. Furthermore, there is a trend towards higher abundance of *Nitrospirae* phylum in non-responders to investigational HPV therapeutic vaccine for high-grade squamous intraepithelial lesions of the cervix [36]. Loss of spermine oxidase (SMOX) could shift the composition of microbiome including increase *Deferribacteres* in chronic colitis and increase the risk for developing CRC [37].

In contrast, at the genus level, *Lactobacillus*, *Parabacteroides* and *rC4-4* showed a positive correlation with *D-Proline.* Meanwhile, *Parabacteroides* and *rC4-4 also* positive correlation with *4-Trimethylammoniobutanoic acid* and *1-Palmitoylphosphatidylcholine*, while negatively correlating with *Isoleucyl-Valine*. The *Lactobacillus* as a kind of probiotics and the reduction of *Lactobacillus* genus displayed a protective effect against CRC [38, 39]. Meng et al. declared that *Lactobacillus paracasei* L9 (LP) regulates arginine and proline metabolism by altering gut microbial metabolites (D-proline, sarcosine), therby improving the experimental autoimmune neuritis (EAN) model [40]. The *Parabacteroides* genus is a constituent of the core gut microbiota in the human body and plays a critical role in maintaining intestinal health and functionality. *Parabacteroides* has demonstrated a capacity to produce acetate by reducing neutrophil infiltration, thereby alleviating heparinase-aggravated acute pancreatitis [41]. Furthermore, freeze-dried *Parabacteroides distasonis*(Pd) has been shown to suppress the occurrence of colorectal tumors in both obese and non-obese mice [42, 43].The genus *rc4-4* belongs to the *Peptococcaceae* family. In comparison to healthy individuals, patients with acute ischemic stroke exhibit a significant dysbiosis in their blood microbiota, characterized by a notable reduction in *rc4-4* abundance [44]. Based on the above, the *Nitrospirae* phylum and *Deferribacteres* phylum may exhibit negative effects in CRC, while CR reduces their abundance and diversity in CRC and is associated with certain metabolites. Conversely, the *Lactobacillus*, *Parabacteroides* and *rc4-4* genus demonstrate opposite effects.

Nevertheless, there are some limitations in our study. We used the HCT116 cell line to establish a colorectal cancer xenograft mouse model because of its rapid growth rate, easy culture, excellent tumorigenicity in nude mice and so on. Only using HCT116 cell line to construct the mouse model, and the insufficient number of mice are the shortcomings of our study. Therefore, using multiple cell lines, increasing the number of animal experiments in mice, and choosing a better way to establish mouse xenograft tumor model as well as orthotopic model of CRC are needed to ensure the reliability and completeness of these findings. Additionally, we did not conduct targeted metabolomics analysis based on the screened metabolites. While betaine has many benefits, there are also studies indicating an increased risk of cardiovascular disease (CVD) associated with betaine and choline intake [45]. But there is still ongoing debate and controversy surrounding this topic [46, 47]. Contrary to our findings of increased Parabacteroides abundance in the CR group, other studies have reported a significant decrease in Pd after CR [48]. This suggests that CR may have differential effects on microorganisms in different cellular contexts, which requires further investigation. Moreover, certain studies have demonstrated that fasting can upregulate FDFT1 expression, inhibit AKT/mTOR/HIF1α signaling transduction, and suppress aerobic glycolysis and proliferation in CRC cells [49]. However, severe calorie restriction leads to decreased bacterial abundance and remodeling of the gut microbiota [50]. The proportion and timing of calorie restrictions are also crucial factors to consider.

## Conclusion

In conclusion, our study demonstrate calorie restriction leads to changes in the fecal metabolite composition of CRC mice. Notably, metabolites such as Isoleucyl-Valine, D-Proline, 4-Trimethylammoniobutanoic acid, and 1-Palmitoylphosphatidylcholine exhibit altered expression levels in the CR group. These differentially expressed metabolites are associated with upregulated metabolic pathways, including Arginine and proline metabolism, Valine, leucine and isoleucine biosynthesis, and Choline metabolism in cancer. Moreover, the combined analysis of microbiota and metabolites reveals a correlation between *Lactobacillus* and specific differentially expressed metabolites like D-Proline. Consequently, these metabolites may serve as potential biomarkers for colorectal cancer, contributing to the advancement of therapeutic approaches.

## Data Availability

All data generated or analysed during this study are included in this article. Further enquiries can be directed to the corresponding author.
